# Predictors of outcomes in infants with gastroschisis treated with a preformed silo

**DOI:** 10.1007/s00383-024-05922-7

**Published:** 2024-12-05

**Authors:** Rebecca Lee, Theodore Dassios, Niyi Ade-Ajayi, Mark Davenport, Ann Hickey, Anne Greenough

**Affiliations:** 1 Department of Women and Children’s Health, School of Life Course Sciences, Faculty of Life Sciences and Medicine, King’s College London, London, UK; 2 Neonatal Intensive Care Centre, King’s College Hospital NHS Foundation Trust, 4th floor Golden Jubilee Wing, Denmark Hill, London, SE5 9RS UK; 3 Department of Paediatric Surgery, King’s College Hospital NHS Foundation Trust, London, UK; 4 Department of Neonatology, Children’s Health Ireland at Crumlin and Temple Street, Dublin, Ireland

**Keywords:** Anterior wall defects, Gastroschisis, Surgical intervention, Preformed silo, Outcomes

## Abstract

**Purpose:**

To describe the outcomes of infants with gastroschisis treated with a preformed silo (PFS) and determine whether routinely measured early physiological parameters, sepsis (blood culture positive), gastroschisis complexity or location of birth influenced the length of stay (LOS) and duration of parenteral nutrition (PN).

**Methods:**

Infants cared for in a tertiary surgical neonatal intensive care unit during a 10-year period were identified.

**Results:**

Seventy-seven infants were assessed [median gestational age 36 + 6 (IQR 35 + 3 to 38 + 0)] weeks. All survived; 82% were inborn. The median LOS was 37 (IQR 28–76.5) days and duration of PN was 28 (IQR 21–53) days. In the first 72 h, the worst median lactate, base excess and ‘toe-core’ gap were 4.2 (IQR 3.0–5.8) mmol/l, -7.0 (IQR − 5.55 to − 9.35), 3.4 (IQR 3.0–4.2) °C respectively. There were no significant correlations between early physiological parameters or place of birth and LOS or PN days, but sepsis (*n* = 18 infants) and complex gastroschisis (*n* = 13 infants) were associated with an increased LOS and PN duration (both *p* < 0.001).

**Conclusions:**

Survival was 100% in infants with gastroschisis who were managed with PFS, sepsis and gastroschisis complexity were associated with a longer hospital stay and duration of parenteral nutrition.

*Level of Evidence (I-V)*: IV.

## Introduction

Gastroschisis (GS) is a frequently occurring congenital malformation with an incidence of approximately 4–5 per 10,000 live births [1]. It requires urgent, specialised neonatal surgical care to achieve survival with minimal morbidity. The method of bowel reduction used varies worldwide [2, 3]. Siting a pre-formed Silo (PFS) enables a gradual, staged reduction of the herniated bowel into the abdominal cavity [4, 5]. Such a technique reduces the risks of visceral-abdominal disproportion, intra-abdominal hypertension, and abdominal compartment syndrome which are associated with primary closure [5, 6]. Indeed, in one observational study, PFS compared to primary closure was associated with lower ventilator pressures, better tissue perfusion and improved early renal function consistent with a reduction in abdominal compartment syndrome [5]. Other studies, however, have demonstrated similar outcomes between the different methods of closure. A retrospective case note review of 156 infants demonstrated no significant differences in the time to full feeds, length of hospital stay (LOS), mortality or morbidity [7]. In addition, an observational study of 566 neonates demonstrated that outcomes were largely similar in infants who underwent immediate closure and those who had a silo placement if this was for less than 5 days [8]. A meta-analysis demonstrated that when PFS rather than primary closure was used as the intended first-line surgical management, there were better short-term outcomes regarding LOS, total parenteral nutrition (TPN) days, days of ventilation and infections rates in studies which had the least selection bias [9]. When, however, all studies were included, primary closure was associated with improved outcomes. Indeed, primary closure in one series was associated with improved outcomes regarding shorter time to initiate enteral feeds and discontinue TPN [10]. It was, therefore, concluded that large prospective studies were needed to definitively determine the best closure technique.

The risk stratification of GS infants to date has mostly focussed on predicting mortality [11, 12] using evidence from observational studies of different surgical practices. The data from four Finnish University Hospitals demonstrated that complex gastroschisis was associated with higher in-hospital mortality, but a risk stratification score was only moderately predictive [13]. Furthermore, in a single centre study, complex gastroschisis was associated with greater days on TPN and LOS [14]. There is, however, little evidence to determine whether pre-closure physiology and management in the immediate hours and days after birth influences outcomes [15]. This lack of data is even more marked in GS infants managed solely via gradual reduction using a PFS.

The aim of our study was to describe the outcomes of infants with gastroschisis treated with PFS as first-line choice of closure. The outcomes included death, sepsis, number of operations, and duration of parenteral nutrition as has been recommended [16]. Furthermore, we investigated whether routinely measured early physiological parameters, sepsis and location of birth (i.e. being out-born from the surgical centre) influenced outcomes. Central line sepsis has been independently associated with mortality [17]. We hypothesised that a metabolic acidosis or poor perfusion status in the first 72 h after birth might indicate bowel compromise and relate to worse clinical outcomes.

## Methods

We used an electronic patient record system, Badgernet Clevermed UK (Badgernet), to identify infants with a gastroschisis during a 10-year period (1st Jan 2008 to 31st Dec 2017). The data were extracted from Badgernet and the medical records. Permission to undertake case note review and data collection was obtained from the local audit lead. The project was registered with the Clinical Governance Department of King’s College Hospital NHS Foundation Trust and the Trust Audit committee (Audit number CH174). The Health Research Authority Toolkit of the National Health System, United Kingdom confirmed that the study would not need regulatory approval by a research ethics committee.

### Physiological parameters

Physiological parameters: serum pH, serum lactate, base excess (BE) and core and peripheral temperatures to calculate the toe-core gap, were collected on admission and the worst values for each parameter up to 72 h after birth were recorded. We also recorded whether infants had a metabolic acidosis: a pH < 7.26 and a BE of − 8.5 mmol/L or greater [18, 19]. pH, lactate and base excess were measured using point of care gas analysers available on the NICU. The core temperatures were measured four hourly using an axillary thermometer. The peripheral skin temperature was monitored continuously by a probe placed on the sole of the baby’s foot. The toe-core temperature difference was calculated as the difference between the axillary temperature and the continuous peripheral temperature reading and was used as a proxy for perfusion.

### Sepsis

Sepsis was defined as blood culture positive associated with either a clinical deterioration and/or raised infection markers and the infant received intravenous antibiotics.

### Outcome measures

The total length of stay (LOS) and the duration of parenteral nutrition (PN) were the outcome measures. The LOS was calculated as days from birth until discharge to the baby’s home. The duration of PN was used as a proxy measure of the duration central lines were in situ as all PN was administered through central lines. Full enteral feeds were defined as tolerating a minimum of 120 ml/kg/day. PN was discontinued at that threshold with the removal of the central line. The enteral feeds continued to be advanced to at least 150 ml/kg/day depending upon the nutritional needs of the infant.

### Data collection

Demographic data were obtained from BadgerNet and the patients’ medical records. The resuscitation and admission information were obtained from the medical case notes over the first 24 h and information entered on the BadgerNet admission record. The intensive care nursing charts were reviewed to obtain all recorded physiological parameters over the first 72 h. Surgical entries in the patient case notes included findings on first examination and description of the closure technique used. The classification of patients into complex or simple gastroschisis was obtained from the surgical entry in the notes and based upon the description of the bowel condition at first surgical assessment and diagnosed as complex if there was at least one of intestinal ischaemia, necrosis, perforation or atresia [11, 20].

### Statistical analysis

Whether the data were normally distributed was assessed using the Kolmogorov–Smirnov test. The data were found to be not normally distributed and, therefore, analysed using non-parametric tests. To determine if differences were statistically significant the Mann–Whitney U test or Chi-squared test were used as appropriate. The Kendall rank correlation coefficient (*r*) was used to assess the association between early physiological parameters and outcomes and multivariate linear regression was used to assess the effect of sepsis and complex gastroschisis on the outcomes. The data were analysed in Excel and SPSS (IBM SPSS Statistics version 25).

## Results

One-hundred infants with gastroschisis were cared for over the 10-year study period, all cases had been diagnosed on antenatal ultrasound examinations. In 16 of these, either the notes were unavailable or there was incomplete information on the physiological parameters and thus they were excluded from the analysis. Seven infants underwent primary closure and were not included in the analysis, all of them survived. The reasons for primary closure were required resection and ileostomy for perforation and necrotic appearance of the bowel, defect too small for PFS, closing defect and PFS applied but bowel appearance deteriorated. Seventy-seven infants were managed with staged reduction using a PFS, all of them survived. Their median birth weight was 2.458 (IQR 1.993–2.757) kg; 51% of the cohort had a birth weight < 2.5 kg. Their median gestational age was 36 + 6 (IQR 35 + 3 to 38 + 0) weeks; 36% were born before 36 weeks of gestation. Eighty-two percent of infants were born in the surgical centre. Of those born elsewhere, one was born at home and presented to their local hospital via ambulance before transfer. All babies were transported via specialist neonatal transfer teams, the mean time to arrival was 5 h.

Ninety percent of babies required mechanical ventilation at some stage during their admission, median days of ventilation was 4 (IQR 2–8). The median LOS was 37 (IQR 28–76.5) days and the duration of PN was 28 (IQR 21–53) days (Table 1). The median duration of supplementary oxygen was 1 (IQR 0–3) days and none of the infants required supplementary oxygen at home. Eighteen infants had at least one episode of sepsis, four had two or more episodes. The blood culture results are presented in Table 2. Five infants had NEC, four were managed conservatively and one, post gastroschisis closure, required a hemicolectomy.

**Table 1:** Demographics and outcomes

Male gender	38 (49)
Gestational age	36 + 6 (35 + 3 to 38 + 0)
Birth weight (g)	2458 (1993–2757)
Birth OFC (cm)	32.4 (30.7–33.5)
Caesarean Section	27 (35)
Required IPPV at birth	24 (31)
In-born at surgical centre	63 (82)
Out-born time to arrival (hrs)	5 (4.0–5.6)
Complex gastroschisis	13 (17)
Days to complete closure	6 (5–8)
Requiring two or more surgical procedures	14(18)
Requiring mechanical ventilation (at any time)	70 (90)
Days ventilated	4 (2–8)
Fluid (ml/kg) in first 4 h	30.5 (23–40)
Deaths	0
Length of stay (days)	37.0 (28.5–76.5)
PN (days)	28.0 (21– 53)
*Physiological parameters in the first 72 h*	
Worst pH	7.24 (7.18–7.29)
Worst lactate (mmol/L)	4.2 (3–5.8)
Worst BE	− 7.0 (−5.55 to − 9.35)
Greatest toe—core gap (^o^Celsius)	3.4 (3–4.2)
Metabolic acidosis	23 (30)

Data are expressed as median (interquartile range (IQR)) or *n*(%)

*OFC* occipitofrontal circumference,* IPPV* intermittent positive pressure ventilation, *BE* base excess, *NG* nasogastric, *PN* parenteral nutrition

**Table 2:** Blood culture results

Pathogen	Number of cases
Coagulase negative staphylococci	14
Escherichia coli	3
Enterococcus faecalis	3
Enterobacter cloacae	1
Pseudomonas aeruginosa	1
Methicillin-sensitive Staphylococcus aureus	1

All babies demonstrated improvement and normalisation of physiological measures by 72 h after birth. There were no significant correlations between physiological parameters and outcomes (PN: worst pH *r* = 0.045; worst lactate *r* = 0.063; greatest BE *r* = 0.052; greatest toe core gap *r* = 0.016 and LOS: worst pH *r* = 0.056; worst lactate r = 0.069; greatest BE *r* = 0.030; greatest toe core gap *r* = 0.015). There were no significant differences between infants who had or had not a metabolic acidosis and PN days (*p* = 0.452) or LOS (*p* = 0.624).

There were no statistically significant differences in the demographics between those with a simple or complex gastroschisis, nor in the results of their early physiological parameter results. Infants with a complex gastroschisis had a delay in time to complete closure; median 7 days (IQR 6–9 days) compared to those with a simple gastroschisis; median 6 days (IQR 5–7 days) (*p* = 0.027). Complex gastroschisis infants were more likely to require two or more operations (69%) compared to those with simple gastroschisis (8%). There was a significant difference in the duration of PN between the groups (Table 3). Two infants required PN on discharge to the surgical ward, one had an ileal atresia with an end to end anastomosis, microcolon and stomal formation on day 68 and the other had significant small bowel resection. Both failed to establish enteral feeds by day 90 and classified as short gut syndrome. Seven other infants also failed to establish full enteral feeds by day 90 and were still receiving PN. In the majority of cases, this was due to dysmotility resulting in large volume NG aspirates. Three had discrepancy in the calibre of the bowel with dilated duodenum and had early small bowel resection resulting in a mechanical obstructive picture. The complex gastroschisis infants required a hospital stay three times longer than the simple gastroschisis babies. The infants with complex gastroschisis were more likely to have an episode of sepsis (42%) compared to those with simple gastroschisis (17%) (*p* = 0.033).

**Table 3:** Demographics and outcomes according to complexity status

	Simple *n* = 64	Complex *n* = 13	p value
Gestational age	36 + 6 (35 + 3 to 38 + 1)	36 + 6 (35 + 1 to 37 + 6)	0.582
Birth weight (g)	2475 (1952–2769)	2240 (2041–2711)	0.501
First pH	7.26 (7.18–7.30)	7.22 (7.11–7.27)	0.344
Worst pH in 72 h	7.24 (7.18–7.29)	7.21 (7.09–7.29)	0.487
Worst lactate in 72 h	4.1 (2.9–5.8)	4.8 (3.1–7.5)	0.467
Worst BE in 72 h	− 6.65 ( − 8.75 to − 5.42)	− 8.8 ( − 12.6 to 6.3)	0.06
Greatest toe–core gap in 72 h	3.4 (3.0–4.2)	3.4 (2.4–4.5)	0.764
Complete closure day	6 (5–7)	7 (6–9)	0.027
Out- born from KCH	15 (22)	1 (6)	0.282
Culture positive sepsis	12 (17)	7 (43)	0.033
PN days	25.0 (20.0–33.7)	83.0 (52.0–138.0)	< 0.001
LOS	34.0 (26.0–52.00)	110.0 (82.0–185.0)	< 0.001

Data expressed as Median (interquartile range (IQR)) or *n* = (%)

*LOS* Length of stay. *BE* Base excess.* PN* Parenteral nutrition

There were no statistical differences in the demographics between the groups who did or did not develop sepsis (Table 4). In the simple gastroschisis group, sepsis was associated with significantly longer LOS and duration of TPN (both *p* < 0.001). The location of birth did not result in significant differences regarding development of sepsis (*p* = 0.499), whether a baby had a complex gastroschisis (*p* = 0.282), the duration of TPN (*p* = 0.293) or the LOS (*p* = 0.306) (Table 4).

**Table 4:** Demographics and outcomes by sepsis status

	No sepsis	Sepsis	p value
	*n* = 59	*n* = 18	
Gestational age	37 + 0 (35 + 4 to 38 + 1)	36 + 0 (35 + 2 to 37 + 3)	0.159
Birth weight (g)	2500 (1955–2792)	2179 (2012–2582)	0.125
First pH	7.24 (7.17–7.30)	7.25 (7.21–7.29)	0.431
Worst pH in 72 h	7.22 (7.16–7.29)	7.26 (7.19–7.29)	0.208
Worst lactate in 72 h (mmol/l)	4.4 (3.0–6.4)	3.5 (2.8–5.3)	0.287
Worst BE in 72 h	− 7.1 ( − 9.6 to − 5.6)	− 6.3 ( − 8.8 to − 5.1)	0.420
Greatest toe – core gap in 72 h	3.4 (3.0–4.2)	3.5 (2.7 to 4.3)	0.690
Complete closure day	6 (5–8)	7.0 (5.75–7.25)	0.582
PN days	25.0 (20.0–31.0)	71.5 (52.5–112.5)	< 0.001
Total LOS	34.0 (26.0–51.0)	95.5 (71.7–117.0)	< 0.001
*Simple gastroschisis only*			
	*n* = 52	*n* = 12	
PN days	24.0 (20.0–29.0)	60.0 (34.5–80.7)	< 0.001
LOS	32.0 (26.0–40.0)	76.5 (52.7–95.7)	< 0.001

Data expressed as Median (interquartile range (IQR))

*LOS* Length of Stay. *BE* Base Excess. *PN* Parenteral Nutrition

Multivariate linear regression analysis demonstrated significant relationships between complex gastroschisis and the incidence of culture positive sepsis and LOS (*p* < 0.001).

## Discussion

All the infants with gastroschisis treated with a preformed silo and gradual bowel reduction survived, as did the seven infants in the same time period who had a primary closure, thus we do not feel there was a hidden mortality. This compares very favourably with the results from low and middle income countries (LMICs) and further argues for collaboration to improve access to high quality neonatal surgical care in LMICs.

Both sepsis and complexity of the gastroschisis, but not abnormal physiological parameters in the first 72 h, were associated with a longer LOS and duration of PN. All the infants were born relatively mature and this has been found to be associated with better outcomes [10, 21]. The pulmonary outcomes of this cohort were good as indicated by the length of ventilation and supplementary oxygen and none of the infants required home oxygen. Respiratory compromise can occur following primary closure [22], but may also be due to pulmonary hypoplasia [23–25].

The severity of illness and risk-adjusted scores of morbidity and mortality based upon infant characteristics and physiological parameters have been used widely in neonatal medicine. The majority, however, have been derived and validated from medical patients, excluding those with congenital malformations or requiring surgery soon after birth [26, 27]. Despite this, they have been used in neonatal surgical populations. A multicentre retrospective study in Canada [28] reported that the Score for Neonatal Acute Physiology-II (SNAP-II) was a predictor of days of TPN and LOS in infants with gastroschisis. In that analysis, however, there was no stratification into complex or simple gastroschisis groups which would be expected to have differing outcomes. The majority of the highest scoring and most physiologically unstable babies underwent an attempted urgent primary closure within 6 h after birth perhaps suggesting they were presenting with greater bowel compromise. The measures which make up the SNAP-II score demonstrated an association with outcomes in that GS cohort, but they are not readily reproducible in the general population of babies born with GS world-wide, who are often near term, born without other system malformation or compromise and do not require invasive arterial line monitoring. The GS infants who undergo a staged closure with PFS, often do not require intubation and ventilation, other than for sedation and analgesia at the time of skin closure which is different to babies who undergo primary closure. There is no evidence of physiological prediction scores being used in cohorts of gastroschisis patients managed with PFS.

Early metabolic acidosis was seen frequently in our cohort. This may be a result of either intestinal ischemia and inflammation due to constriction of the bowel or exposure of the bowel to amniotic fluid in utero. It could also result from fluid imbalance and hypovolemia as a result of losses from the exposed bowel and third space losses. The difficulties in labour or placental compromise may also be confounders. Despite the frequency of this presentation, we found that all infants in our cohort attained physiological stability and normalisation of parameters within 72 h. Fluid management in babies who undergo a staged reduction via PFS where losses are relatively easily measurable over the early days following birth has not been widely reported, but may have a beneficial effect in attaining the stability we report. The toe-core temperature difference is a measure of peripheral perfusion and circulatory adequacy in adult, paediatric and neonatal intensive care settings [29]. The normal toe-core gap in neonates is between 1 and 2 °C [30]. Although, many of the infants had a toe-core gap outside this range this had normalised by 72 h and hence this may explain why we did not find an association with outcomes.

Blood culture positive sepsis had a deleterious effect on outcomes in both the complex and simple GS groups in keeping with other reported findings [15, 31, 32]. Seventeen percent of the simple GS group developed culture positive sepsis which was associated with a significantly increased LOS. Efforts should be focussed to improve care and reduce harm by identifying modifiable factors to reduce culture positive sepsis [33, 34] alongside regular surveillance of local infectious complications in this group of infants.

This study has a number of strengths and some limitations. Although this is a retrospective study, the efforts were made to ensure the accuracy of the surgical stratification of cases and completion of collection of physiological parameters and microbiological information for each infant. The novelty of our study lies in the focus on infants treated with a PFS and the exploration of early physiological parameters as possible predictors of later outcomes. The study population numbers were limited by the availability of complete patient case notes, but we were still able to report the outcomes of 77 infants who had a PFS. We report a single centre’s experience in which all infants received uniform medical and surgical management enhancing the validity of our findings.

In conclusion, infants with gastroschisis managed with a preformed silo had 100% survival. Sepsis and complex gastroschisis, but not early physiological parameters, were associated with a longer stay or duration of parenteral nutrition.

## Previous communication

Results presented at the 3rd Congress of Joint European Neonatal Societies, Sept 2019.

## Data Availability

No datasets were generated or analysed during the current study.

## Abbreviations

BE: Base excess
GS: Gastroschisis
LOS: Length of stay
PFS: Preformed silo
TPN: Total parenteral nutrition

## References

[1] Bhat V, Moront M, Bhandari V (2020) Gastroschisis: a state of the art review. Children (Basel) 177:302. 10.3390/children7120302 10.3390/children7120302 PMC7765881 33348575

[2] Owen A, Marven S, Johnson P et al (2010) Gastroschisis: a national cohort study to describe contemporary surgical strategies and outcomes. J Pediatr Surg 45:1808–1816. 10.1016/j.jpedsurg.2010.01.036 20850625 10.1016/j.jpedsurg.2010.01.036

[3] Zani A, Ruttenstock E, Davenport M et al (2012) Is there unity in Europe? First survey of EUPSA delegates on the management of gastroschisis. Eur J Pediatr Surg 23:19–24. 10.1055/s-0032-1326954 23093440 10.1055/s-0032-1326954

[4] Fischer J, Chun K, Moores D et al (1995) Gastroschisis: a simple technique for staged silo closure. J Pediatr Surg 30:1169–1171. 10.1016/0022-3468(95)90014-4 7472975 10.1016/0022-3468(95)90014-4

[5] Allotey J, Davenport M, Njere I et al (2007) Benefit of preformed silos in the management of gastroschisis. Pediatr Surg Int 23:1065–1069. 10.1007/s00383-007-2004-9 17694400 10.1007/s00383-007-2004-9

[6] Yaster M, Buck JR, Dudgeon DL et al (1988) Hemodynamic effects of primary closure of omphalocele/gastroschisis in human newborns. Anesthesiology 69:84–88. 10.1097/00000542-198807000-00012 2968772 10.1097/00000542-198807000-00012

[7] Charlesworth P, Akkinola I, Hammerton C et al (2014) Preformed silos versus traditional abdominal wall closure in gastroschisis: 163 infants at a single institution. Eur J Pediatr Surg 24:88–93. 10.1055/s-0033-1357755 24163195 10.1055/s-0033-1357755

[8] Hawkins R, Raymond S, St. Peter D et al (2020) Immediate versus silo closure for gastroschisis: results of a large multicenter study. J Pediatr Sur 55:1280–1285. 10.1016/j.jpedsurg.2019.08.002 10.1016/j.jpedsurg.2019.08.002 PMC7731615 31472924

[9] Kunz S, Tieder J, Whitlock K et al (2013) Primary fascial closure versus staged closure with silo in patients with gastroschisis: a meta-analysis. J Pediatr Sur 48:845–857. 10.1016/j.jpedsurg.2013.01.020 10.1016/j.jpedsurg.2013.01.020 PMC4103994 23583145

[10] Palatnik A, Loichinger M, Wagner A et al (2020) The association between gestational age at delivery, closure type and perinatal outcomes in neonates with isolated gastroschisis. J Matern Fetal Neonatal Med 33:1393–1399. 10.1080/14767058.2018.1519538 30173575 10.1080/14767058.2018.1519538

[11] Molik K, Gingalewski C, West K et al (2001) Gastroschisis: a plea for risk categorization. J Pediatr Surg 36:51–55. 10.1053/jpsu.2001.20004 11150437 10.1053/jpsu.2001.20004

[12] Arnold M, Chang D, Nabasweesi R et al (2007) Development and validation of a risk stratification index to predict death in gastroschisis. J Pediatr Surg 42:950–956. 10.1016/j.jpedsurg.2007.01.028 17560201 10.1016/j.jpedsurg.2007.01.028

[13] Tauriainen A, Raitio A, Tauriainen T et al (2022) Comparison of three risk stratification scores in gastroschisis neonates: gastroschisis prognostic score, gastroschisis risk stratification index and complex gastroschisis. Pediatr Surg Int 38:1377–1383. 10.1007/s00383-022-05180-5 35881242 10.1007/s00383-022-05180-5 PMC9458559

[14] Melov SJ, Tsang I, Cohen R et al (2018) Complexity of gastroschisis predicts outcome: epidemiology and experience in an Australian tertiary centre. BMC Pregnancy Childbirth 18:1–9. 10.1186/s12884-018-1867-1 29890949 10.1186/s12884-018-1867-1 PMC5996507

[15] Chen L, Moore J, Horsager-Boehrer R et al (2017) Intrinsic risk for poor outcome in neonates born with gastroschisis: a systemic review. JSM Pediatr Surg 1:1003–1013. 10.47739/2578-3149/1003

[16] Allin BS, Hall NJ, Ross AR et al (2019) Development of a gastroschisis core outcome set. Arch Dis Child Fetal Neonatal Ed 104:F76-82. 10.1136/archdischild-2017-314560 29540463 10.1136/archdischild-2017-314560 PMC6762000

[17] Tauriainen A, Harju S, Raitio A et al (2023) Longitudinal growth of children born with gastroschisis or omphalocele. Eur J Pediatr 182:5615–5623. 10.1007/s00431-023-05217-4 37819418 10.1007/s00431-023-05217-4 PMC10746581

[18] El-Naggar W, Almudeer A, Vincer M et al (2017) Preoperative metabolic acidosis in infants with gastroschisis. J Neonatal Perinatal Med 10:307–311. 10.3233/NPM-16128 28854513 10.3233/NPM-16128

[19] Malan A, Evans A, Heese H (1965) Serial acid-base determinations in normal premature and full-term infants during the first 72 hours of life. Arch Dis Child 40:645–650. 10.1136/adc.40.214.645 5847252 10.1136/adc.40.214.645 PMC2019493

[20] Youssef F, Laberge J, Puligandla P et al (2017) Determinants of outcomes in patients with simple gastroschisis. J Pediatr Surg 52:710–714. 10.1016/j.jpedsurg.2017.01.019 28188037 10.1016/j.jpedsurg.2017.01.019

[21] Overcash RT, DeUgarte DA, Stephenson ML et al (2014) University of California Fetal Consortium. Factors associated with gastroschisis outcomes. Obstet Gynecol 124:551. 10.1097/AOG.0000000000000425 25162255 10.1097/AOG.0000000000000425 PMC4147679

[22] Dimitriou G, Greenough A, Giffin F et al (1996) Temporary impairment of lung function in infants with anterior abdominal wall defects who have undergone surgery. J Pediatr Surg 31:670–672. 10.1016/s0022-3468(96)90671-5 8861478 10.1016/s0022-3468(96)90671-5

[23] Panitch HB (2015) Pulmonary complications of abdominal wall defects. Paediatr Respir Rev 16:11–17. 10.1016/j.prrv.2014.10.004 25458796 10.1016/j.prrv.2014.10.004

[24] Anyanwu LJ, Ade-Ajayi N, Rolle U (2020) Major abdominal wall defects in the low- and middle-income setting: current status and priorities. Pediatr Surg Int 36:579–590. 10.1007/s00383-020-04638-8 32200405 10.1007/s00383-020-04638-8 PMC7165143

[25] Global PaedSurg Research Collaboration (2021) Mortality from gastrointestinal congenital anomalies at 264 hospitals in 74 low-income, middle-income, and high-income countries: a multicentre, international, prospective cohort study. Lancet 398:325–339. 10.1016/S0140-6736(21)00767-4 34270932 10.1016/S0140-6736(21)00767-4 PMC8314066

[26] Richardson D, Gray J, McCormick M et al (1993) Score for neonatal acute physiology: a physiologic severity index for neonatal intensive care. Pediatrics 91:617–623 8441569

[27] Richardson D, Corcoran J, Escobar G et al (2001) SNAP-II and SNAPPE-II: simplified newborn illness severity and mortality risk scores. J Pediatr 138:92–100. 10.1067/mpd.2001.109608 11148519 10.1067/mpd.2001.109608

[28] Mills J, Lin Y, MacNab Y et al (2010) Perinatal predictors of outcome in gastroschisis. J Perinatol 30:809–813. 10.1038/jp.2010.43 20357809 10.1038/jp.2010.43

[29] Schey B, Williams D, Bucknall T (2010) Skin temperature and core-peripheral temperature gradient as markers of hemodynamic status in critically ill patients: a review. Heart Lung 39:27–40. 10.1016/j.hrtlng.2009.04.002 20109984 10.1016/j.hrtlng.2009.04.002

[30] Waldron S, MacKinnon R (2007) Neonatal thermoregulation. Infant 3:101–104

[31] Khalil B, Baath M, Baillie K et al (2008) Infections in gastroschisis: organisms and factors. Padiatr Surg. 43:2208–2212. 10.1007/s00383-008-2210-0 10.1007/s00383-008-2210-0 18668249

[32] Baird R, Puligandla P, Skarsgard E et al (2012) Infectious complications in the management of gastroschisis. Pediatr Surg Int 28:399–404. 10.1007/s00383-011-3038-6 22159577 10.1007/s00383-011-3038-6

[33] Neill S, Haithcock S, Smith P et al (2016) Sustained reduction in bloodstream infections in infants at a large tertiary care neonatal intensive care unit. Adv Neonatal Care 16:52–59. 10.1097/ANC.0000000000000164 25915573 10.1097/ANC.0000000000000164 PMC4619157

[34] Fisher D, Cochran K, Provost L et al (2013) Reducing central line associated bloodstream infections in North Carolina NICUs. Pediatrics 132:1664–1671. 10.1542/peds.2013-2000 10.1542/peds.2013-2000 24249819

